# Family Features of Social Withdrawal Syndrome (Hikikomori)

**DOI:** 10.3389/fpsyt.2020.00138

**Published:** 2020-03-02

**Authors:** Ángeles Malagón-Amor, Luis Miguel Martín-López, David Córcoles, Anna González, Magda Bellsolà, Alan R. Teo, Antoni Bulbena, Víctor Pérez, Daniel Bergé

**Affiliations:** ^1^Institut de Neuropsiquiatria i Addiccions (INAD), Hospital del Mar, Barcelona, Spain; ^2^Department of Psychiatry and Forensic Medicine, Autonomous University of Barcelona, Barcelona, Spain; ^3^Centro de Investigación Biomédica en Red de Salud Mental, CIBERSAM G21, Madrid, Spain; ^4^Hospital del Mar Medical Research Institute (IMIM), Barcelona, Spain; ^5^Center to Improve Veteran Involvement in Care (CIVIC), VA Portland Health Care System, Portland, OR, United States; ^6^Department of Psychiatry, School of Medicine, Oregon Health & Science University, Portland, OR, United States; ^7^School of Public Health, Oregon Health & Science University and Portland State University, Portland, OR, United States

**Keywords:** hikikomori, social isolation, social withdrawal, family factors, family psychiatric history, home treatment, dysfunctional family dynamics, childhood maltreatment

## Abstract

**Background:** Family may play an important role in the origin, maintenance, and treatment of people with social withdrawal. The aim of this study is to analyze family factors related to social withdrawal syndrome.

**Methods:** Socio-demographic, clinical, and family data, including family psychiatric history, dysfunctional family dynamics, and history of family abuse were analyzed in 190 cases of social withdrawal with a minimum duration of 6 months that started an at-home treatment program. Data were analyzed at baseline and at 12 months.

**Results:** In 36 cases (18%) neither the patient nor the family allowed at home evaluation and treatment by the Crisis Resolution Home Treatment (CRHT) team. Patients had high rates of dysfunctional family dynamics (*n* = 115, 61.5%), and family psychiatric history (*n* = 113, 59.3%), especially maternal affective (*n* = 22, 42.9%), and anxiety disorders (*n* = 11 20.4%). There was a non-negligible percentage of family maltreatment in childhood (*n* = 35, 20.7%) and single-parent families (*n* = 66, 37.8%). Most of the cases lived with their families (*n* = 135, 86%), had higher family collaboration in the therapeutic plan (*n* = 97, 51.9%) and families were the ones to detect patient isolation and call for help (*n* = 140, 73.7%). Higher social withdrawal severity (as defined by at least one of: early age of onset, no family collaboration, lack of insight, higher CGSI score, and higher Zarit score), was associated with family psychiatric history, dysfunctional family dynamics, and family abuse history. All of these predictive variables were highly correlated one to each other.

**Conclusions:** There is a high frequency of family psychiatric history, dysfunctional family dynamics, and traumatic events in childhood (family maltreatment), and these factors are closely interrelated, highlighting the potential role of family in the development and maintenance of social withdrawal.

## Introduction

Social withdrawal syndrome was first described in Japan as hikikomori, and defined as the state of confining oneself to one's house for more than 6 months and strictly limiting communication with others (1). In recent years its existence has been described in other countries and cultures, noting that other factors besides Japanese culture may also influence its origin and development (2–4).

We have previously assessed which factors determine withdrawal relapse or persistence (3). Among them, some factors, such as age or comorbid diagnostics, may result in being difficult to modify. However, intensive treatment care, which showed a positive effect on prognosis (3), could be extended with the collaboration of different settings and familial environment.

More than half of the patients affected by social withdrawal maintain a relationship with their families (3, 5), which opens the door for one of the few opportunities for intervention and reinforces the relevance of studying family factors in social withdrawal. However, to our knowledge, few studies have reported data regarding family factors in relation to onset, persistence, or relapse in social withdrawal (6–9).

Family factors that have been identified with the appearance of social isolation include: insecure attachment (10), death of a family member (11), nuclear family without extended family support (12–14), fragmented family (15), dysfunctional family and parenting dynamics (8, 16–18), as well as emotional neglect and child abuse (19). A higher risk has been observed in families with a high economic level, as well as those with a high parental educational level (6). It has been described that some parents who do not know how to start a conversation or worry about their children (8) may not teach their children empathy, how to establish trusting relationships with others, and how to engage in healthy communications (20). Other features, such as traumatic childhood experience and family maltreatment history, have been described as risk factors for developing social withdrawal syndrome (6, 12, 18, 19).

In relation to dysfunctional family dynamics, hikikomori in Japan has been related to the concept of “*amae*” (21), which describes the Japanese dependent behavior in which a person implores, or alternatively acts with selfishness and indulgence, knowing that the caregiver will forgive them. Traditionally, in Asian societies, this is a frequent phenomenon that begins in childhood, generating a family dynamic in which the child uses manipulation to gain parental care, chronifying themselves in time, and thus, becoming economically dependent upon their parents (17, 22, 23). Adding to this phenomenon, the current generation of Japanese young adults has experienced a decrease in desire and motivation (18). In addition, the economic comfort afforded by Japanese families has led to a decrease in the value of work. Finally, it has been pointed out that parents are less strict in the upbringing of their children (24). In Western culture there is no word equivalent to “*amae*,” which may be related to a lower prevalence, although it may also exist.

In relation to the possible influence of parental psychopathology as a risk factor, there are few studies. Panic disorder in the mother has been described as a risk factor (6), suggesting that this association could be attributable to parenting behavior that reinforces the patient's anxiety and avoidant coping strategy. In our research setting, our team described a high frequency of family psychiatric history, mostly maternal affective, and anxiety disorders. Fathers were more likely to have psychotic and drug use disorders (2); however, no other studies have investigated family history by separating the two parents.

In addition, it is undoubted that the family plays a very important role in the detection and treatment of the social withdrawal of their children (8, 23), as they usually alert health services about the situation and ask for help. However, due to prejudice and lack of knowledge, in many cases family members are unable to intervene at all, and the socially withdrawn person tends to hide for many years without seeking help (4). That is why, after the initial consultation, the first step is to alleviate the psychological burden on the parents themselves, support them and relieve their feelings of despair and self-condemnation (7, 8), perform family psychoeducation (4), and facilitate understanding and acceptance of the diagnosis and treatment of social withdrawal. It is also relevant for early detection in relatives or descendants of people with mental illness (2).

All of these aspects lead to the importance of the role of family and hereditary factors in social withdrawal, to understand its influence on the origin of the syndrome, and to apply this knowledge in early detection and treatment, both at the individual and family levels. Therefore, the objective of this study is the analysis of family factors related to social withdrawal and its evolution.

## Materials and Methods

### Participants

The participants were 190 subjects with social withdrawal and their families, who were attended by the Crisis Resolution Home Treatment (CRHT) program from 2008 to 2014 in Barcelona (Spain). The diagnostic inclusion criteria were (25–28): (1) spend most of the day and almost every day at home; (2) avoid social situations, such as attending school or going to work; (3) avoid social relationships, such as friendships or contacts with family members; (4) discomfort or significant deterioration due to social isolation; (5) minimum duration of 6 months. These symptoms should be primary and predominant over any other symptoms, in case there were others. The exclusion criteria were subjects with diagnosed cognitive disorders, such as dementia, drug dependence without other comorbid psychiatric disorders, age younger than 12 years old, and subjects for whom the only treatment option was involuntary inpatient therapy. Diagnosis was made by the psychiatrists of the CRHT team through clinical evaluation.

### Home Visitation Program

The CRHT team comprised two psychiatrists and two nurses. The target population were patients with severe mental disorders disengaged from outpatient monitoring, and people with no psychiatric history who presented behavioral disorders suggestive of mental disorders. Cases were referred to the CRHT by social workers, primary and psychiatry outpatient teams, and/or psychiatric emergency services. When a case was referred to the CRHT, a first interview was performed with the family or caregiver, if it existed, to collect the medical and psychiatric history, socio-demographic data, determine which was the clinical situation, and coordinate the home visit. After, once at home, the diagnostic approach was made and pharmacological treatment was prescribed if necessary. Several home visits were performed until clinical stabilization. The mean number of visits was 4, with a wide range, from 1 to 21 visits. A review of the previous articles will provide more information on the operation of the equipment (2, 3). When CRHT home treatment was completed, cases were referred to the most appropriate device in an individualized manner: outpatient psychiatric or medical center, hospitalization, or others. Following this referral, the clinical condition and situation of isolation was evaluated by contacting the mental health team currently in charge of the subject at 4, 8, and 12 months after referral.

### Measurement Instruments

All cases were prospectively studied according to a routine computerized protocol that included demographic and clinical information. Socio-demographic data included age, gender, social network, and living situation. The latter included screening for dysfunctional family relationships. A dysfunctional family was defined as one whose interrelationships serve to detract from, rather than promote, the emotional and physical health and well-being of its members, with continuous conflict and instability, and with traits such as poor communication, excessive control, perfectionism, lack of empathy, and excessive criticism. The degree of family collaboration in the therapeutic plan was also evaluated. Clinical characteristics included referral source, family psychiatric history, and personal psychiatric history. This included medical history, family abuse history in childhood, and previous contact with any outpatient-type mental health service. The socially withdrawn period and the age at onset of social withdrawal were also recorded. The patient diagnoses were evaluated using the DSM-IV-TR criteria, grouping the major mental disorders into six categories: psychotic, affective, anxiety, drug abuse, personality, and other Axis I diagnoses. The service to which the CRHT referred the case after follow-up was also recorded. Illness severity was assessed using the Spanish version of the Severity of Psychiatric Illness (SPI) scale (29, 30). Subjects were also evaluated using the Global Assessment of Functioning (GAF) (31, 32), the Clinical Global Impressions Scale (33, 34) and the World Health Organization Disability Assessment (WHO/DAS) (35) to measure functioning, the Zarit Burden Interview (36) to assess caregiver burden, and the Scale of Unawareness of Mental Disorder (SUMD) (37) to evaluate insight. Internet addiction was clinically evaluated using the diagnosis criteria more widely accepted (38, 39): (1) excessive Internet use (compulsive striving for Internet usage, growing importance of Internet in the system of personal values, (2) withdrawal symptoms (mood swings like anger, depression, and anxiety when Internet is unavailable), (3) tolerance (need for increased use of the Internet to relieve negative emotional symptoms), (4) negative consequences due to Internet use (excessive engagement in Internet use, loss of previous hobbies and entertainments, loss of social relations, educational and sport opportunities, quarrels and lies). Social network was evaluated according to criteria developed by our team based on clinical experience as follows: (1) null relationship, (2) relationship with family with whom we live, (3) relationship with a friend outside the home, and (4) normalized social relation.

An evaluation of the subject's connection to the mental health network at 4, 8, and 12 months after program discharge was performed by contacting the responsible medical service. This assessment included clinical status evaluated using the GAF and WHO/DAS scales, as well as the persistence of social isolation and its severity.

### Statistical Analysis

First, a descriptive analysis of the sample at baseline was performed. A specific descriptive analysis on the diagnostic family data separated by parent was carried out due to previously described high rates of anxiety disorders in mothers of socially isolated subjects, and the lack of reports of the psychiatric history of fathers.

Second, univariate analysis between baseline family characteristics and severity measures of withdrawal at baseline were calculated using Pearson's correlation coefficient. To this purpose, the variables reflecting severity were age at onset of social withdrawal, social withdrawal time, family collaboration with treatment, null social network, internet addiction, inpatient treatment, and SPI, WHO/DAS, CGI, CGSI, SUMD, ZARIT, and GAF scores. The variables related to family characteristics were family psychiatric history, dysfunctional family dynamics, family maltreatment history, and single-parent family. Those family variables with significant correlation with severity measures were selected for the next step. The same procedure was repeated for severity measures at 12 months adding successful linkage at this point in time. Third, to test the interrelation between the family variables predicting severity, the selected family variables were tested for correlations between each other using chi-square test to test association between categorical variables. Then, to determine the level of clustering within this set of family variables, the selected variables were introduced in two separate hierarchical clustering analyses: first, the subjects' dataset for the selected variables was converted to a Euclidean distance matrix. This matrix was entered in a clustering model using averages as the grouping criteria. The same procedure was repeated for the selected variables, except that the distance matrix was created subtracting the square of the correlation matrix to one. The two resulting dendrograms were plotted one to each other to visualize the correspondence of the grouping of subjects and variables. The subjects' dendrogram was divided into as many groups as the subjective visualization of the dendrogram suggested. Finally, to control the possible confounding effect of gender and age in the relation between family factors and severity of isolation, we computed a multivariate regression analysis. To this purpose, among the initial pool of variables of severity of social withdrawal, we selected those that showed significant correlation with family factors in the univariate analysis (age at onset of social withdrawal, family collaboration with treatment, CGSI, SUMD, ZARIT) and entered them as dependent variables. As independent variables, we entered the previously mentioned family factors (family psychiatric history, dysfunctional family dynamics, family maltreatment history, and single-parent family), and age and sex.

Analyses were conducted using SPSS 24 (SPSS, Chicago, IL, USA), except for cluster analysis and multivariate analysis that were conducted using R (RStudio, v. 1.1.423—^©^ 2009–2018 RStudio, Inc).

## Results

**Figure 1:**
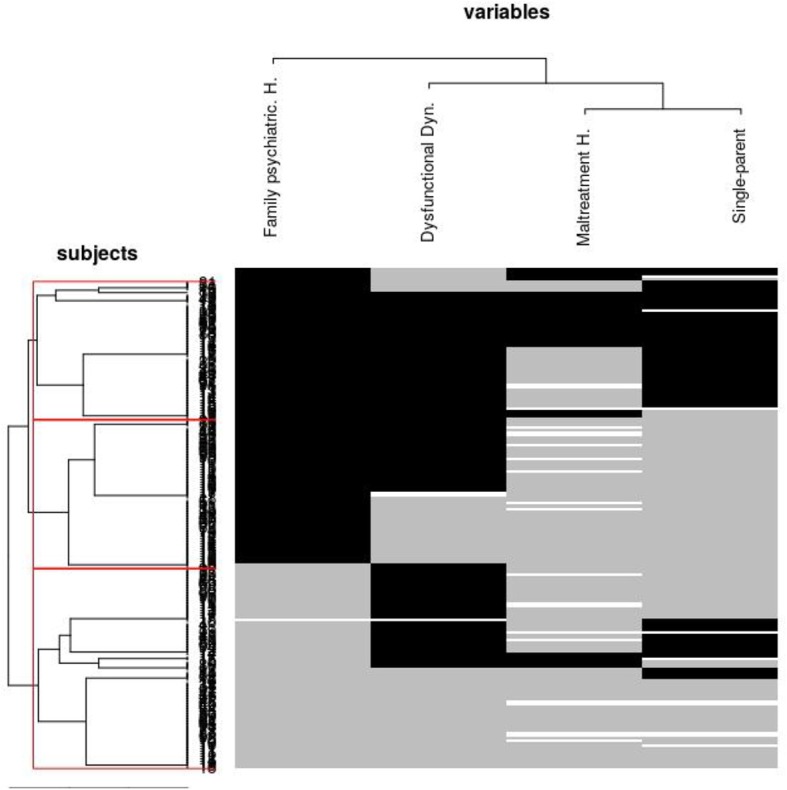
Dendrogram showing hierarchical cluster analysis of those family variables most related to social withdrawal severity and subjects. Black color in the matrix represents a dichotomous “yes” value for the variable. Gray color represents “not present” for the variable. White color represents missing data. A small proportion of subjects endorsed for all the family variables that predicted severity of isolation (black stripe at the bottom of the plot). A thicker proportion of subjects did not endorse for many of the selected variables (subjects middle group), and finally, another significant proportion of subjects endorsed for almost all the variables. Family variables were clustered in the following order Single-parent family and Maltreatment history -> Dysfunctional family dynamics -> Family psychiatric history.

The global sociodemographic and clinical characteristics of cases treated at home are shown in Table 1. A more detailed description of the socio-demographic data of this sample can be found in our previous work (2, 3). In 36 cases (18%), neither the patient nor the family allowed the CRHT evaluation and treatment at home. Each of the family variables was significantly related to at least one of the severity variables as described below (see Table 2). These family variables were strongly interrelated (see Table 3). The difference between the ultrametric distances of the cluster analysis and the original distances was 0.0007 for the cluster analysis of the variables, and 0.0875 for the cluster analysis of the subjects, indicating good cluster modeling. Figure 1 shows the dendrogram plot for the selected variables and subjects.

**Table 1:** Global sociodemographic and clinical characteristics of social withdrawal cases attended by CRHT.

**Variables**	**Total social withdrawal cases (*n* = 190) *n* (%)**
Gender, Male	137 (72.1)
Age, mean (*SD*)	39.1 (18.1)
Isolation period, months, mean (*SD*)	38.1 (52.3)
Age at onset of isolation, mean (*SD*)	36.2 (17.9)
**WHO DETECTS SOCIAL ISOLATION**	
- Family (vs. medical services)	140 (73.7)
**LIVING SITUATION**	
- Alone	30 (15.8)
- Family	160 (84.2)
- Family of origin	131 (81.8)
- Own family	29 (18.2)
Single-parent family	68 (37.8)
- With mother	64 (94.1)
- With father	4 (5.9)
**EDUCATION LEVEL**	
- Secondary or higher	77 (40.7)
- University	22 (11.6)
**EMPLOYMENT STATUS**	
- Unemployed	117 (61.6)
- Student	18 (9.5)
- Worker	0 (0)
- Old age pensioner	17 (8.9)
- Medical pensioner	32 (16.2)
- Sick leave	6 (3.2)
Dysfunctional family dynamics	115 (61.5)
**FAMILY PSYCHIATRIC HISTORY**	
- First grade	87 (76.9)
- Mother	51 (59.7)
- Father	17 (19.5)
- Brother	19 (20.7)
Second grade	26 (23.1)
Internet addiction	59 (31.4)
**REFERRED SERVICE BY THE CRHT**	
- Medical outpatient team	26 (16.6)
- Psychiatric outpatient team	62 (39.5)
- Hospitalization	48 (30.6)
- Other	21 (13.4)
WHO/DAS total, mean (*SD*)	12,8 (3.7)
GAF initial, mean (*SD*)	40,7 (15)
GAF final, mean (*SD*)	45,7 (16.7)
SUMD Total, mean (*SD*)	10 (4.2)
CGIS, mean (*SD*)	2,5 (1.6)
CGIC, mean (*SD*)	4,1 (1.1)
SPI total, mean (*SD*)	12,6 (4.9)
Zarit total, mean (*SD*)	44,7 (19.8)

**Table 2:** Summary of the univariate analysis between variables of severity of isolation and family factors.

	**Age at onset of isolation**	**Isolation time**	**Family collaboration with treatment**	**Null social network**	**Inpatient treatment**	**SPI score**	**WHO/DAS score**	**SUMD score**	**CGIC score**	**CGIS score**	**Zarit score**	**GAF score**
Family psychiatric history	x											
Single-parent family											x	
Dysfunctional family dynamics	x		x							x		
Family maltreatment history			x									

*x, significant correlation; p < 0.05*.

**Table 3:** Correlations between family variables that predict the severity of social withdrawal.

	**Family maltreatment history**	**Dysfunctional family**	**Single-parent family**	**Family psychiatric history**
	***chi-square***	**dynamics *chi-square***	***chi-square***	***chi-square***
Family maltreatment history	NA	11.515^*^	24.943^*^	9.534^*^
Dysfunctional family dynamics	11.515^*^	NA	20.081^*^	6.506^*^
Single-parent family	24.943^*^	20.081^*^	NA	8.481^*^
Family psychiatric history	9.534^*^	6.506^*^	8.481^*^	NA

* *The Chi-Square statistic is significant at the 0.05 level*.

### Family Psychiatric History

Family psychiatric history is described in Table 1 and separated by gender of the parents in Table 4. In 21 cases, the family psychiatric history was unknown.

**Table 4:** Family psychiatric diagnosis separated by parent of social withdrawal cases attended at home by CRHT.

**Family psychiatric diagnosis**	**Mother**	**Father**	***p***
	***n* (%)**	***n* (%)**	
Drug use disorder	3 (6.1)	5 (31.2)	0.01
Psychotic disorder	6 (12.2)	9 (50)	
Affective disorder	22 (42.9)	2 (12.5)	
Anxiety disorder	11 (20.4)	1 (6.2)	
Personality disorder	3 (6.1)	0 (0)	
Other	6 (12.2)	0	

Having family psychiatric history was significantly associated with younger age at onset (mean age years 31.5 [*SD* 15.3] vs. 40.2 [*SD* 20.1], *p* = 0.002) and higher rates of internet addiction (*n* = 41 [36.9%] vs. *n* = 18 [23.4%], *B* = 0.136, *p* = 0.049) as measures of withdrawal severity. In regard to 12-month variables of severity, this variable was inversely related to linkage at 12 months (*B* = −0.152, *p* = 0.048). Regarding the associations with other family variables, having psychiatric history was positively related to history of family maltreatment (*n* = 29 [28.7%] vs. *n* = 6 [9%], *X-sq* = 9.534, *p* = 0.002), single-parent family (*n* = 50 [45.9%] vs. *n* = 17 [24.3%], *X-sq* = 8.481, *p* = 0.004), and familial dystocia (*n* = 76 [69.1%] vs. *n* = 39 [50.6%], *X-sq* = 6.506, *p* = 0.011).

### Dysfunctional Family Dynamics

The existence of dysfunctional family dynamics was significantly related to some measures of withdrawal severity: younger age at onset of withdrawal (mean age 31.6 [*SD* 16.8] vs. 40.6 [*SD* 18.2], *p* = 0.001), higher internet addiction (*n* = 43 [37.4%] vs. *n* = 16 [22.5%], *B* = 0.149, *p* = 0.034), and less family collaboration (*n* = 39 [33.9%] vs. *n* = 58 [80.6%], *B* = −0.466, *p* < 0.001). Dysfunctional family dynamics was related with other family variables, such as having family psychiatric history (*n* = 76 [66.1%] vs. *n* = 34 [47.2%], *X-sq* = 6.505, *p* = 0.011), single-parent family (*n* = 56 [50.5%] vs. *n* = 11 [16.7%], *X-sq* = 20.081, *p* < 0.001), and family maltreatment history (*n* = 30 [29.7%] vs. *n* = 5 [7.7%], *X-sq* = 11.515, *p* = 0.001). It was also inversely related with linkage at 12 months follow-up (*n* = 10 [26.3%] vs. 0 [0%], *p* = 0.041, *B* = −0.161).

### Family Maltreatment History

Regarding family maltreatment history, statistically significant differences were found in withdrawal severity measures: less family collaboration with treatment (*n* = 11 [31.4%] vs. *n* = 78 [59.6%], *B* = −0.281, *p* = 0.003) and higher Zarit scores (mean 58.3 [*SD* 18.4] vs. mean 41.4 [18.9 *SD*], *p* = 0.048). No significant differences were found in the 12 months follow-up. As regards to other family variables, family maltreatment history was related with having family psychiatric history (*n* = 29 [82.9%] vs. *n* = 72 [54.1%], *X-sq* = 9.534, *p* = 0.002), dysfunctional family dynamics (*n* = 30 [85.7%] vs. *n* = 71 [54.2%], *X-sq* = 11.515, *p* = 0.001, *B* = 0.263) and single-parent family (*n* = 25 [78.1%] vs. *n* = 40 [30.1%], *X-sq* = 24.943, *p* < 0.001).

### Single-Parent Families

In relation to cases from single-parent families, compared to two-parent families, statistically significant differences were found in some severity variables: age at onset of withdrawal (mean 25.9 [*SD* 12.1] vs. mean 38.3 [*SD* 18.3], *p* < 0.001), social withdrawal time (months mean 27.2 [*SD* 28.1] vs. mean 43.7 [*SD* 62.9], *p* = 0.001), higher internet addiction (*n* = 31 [46.3%] vs. *n* = 27 [24.3%], *B* = 0.219, *p* = 0.002), and higher Zarit scores (mean 53.42 [*SD* 19.37] vs. mean 39.06 [*SD* 19.11], *B* = 14.361, *p* = 0.055). Regarding other family variables, there was more frequent history of family abuse (*n* = 25 [38.5%] vs. *n* = 7 [7%], *X-sq* = 24.943, *p* < 0.001), higher family dystocia (*n* = 56 [83.6%] vs. *n* = 55 [50%], *X-sq* = 20.081, *p* < 0.001) and less family psychiatric history (*n* = 50 [45.9%] vs. *n* = 59 [54.1%], *X-sq* = 8.481, *p* = 0.004). No significant differences were found in the 12 months follow-up.

No other statistically significant differences were found between variables related to family characteristics and sociodemographic, clinical, and severity variables, and variables about follow-up for 12 months.

The multivariate regression analysis controlling for age and sex revealed a non-significant effect of sex (*F* = 0.7, *p* =0.634), a significant effect of age (*F* = 224.4, *p* < 0.001). Dysfunctional family dynamics (*F* = 6.1, *p* =0.013) and being single-parent family (*F* = 4.8, *p* = 0.025) remained significant to predict severity of social withdrawal, while family maltreatment history reached only trend level (*F* = 3.1, *p* = 0.078) and family psychiatric history was non-significant (*F* = 0.6, *p* = 0.684).

Twenty-eight subject (out of 162) had at least one missing value in the variables describing the four selected family factors and thus, they were not entered in the multivariate analysis. In the univariate analysis, all subjects with available data for each analysis were used. Subjects with missing values did not differ with the rest of subjects in terms of CGIS (mean = 4.0 vs. 4.2, *p* = 0.616), GAF at baseline (37.5 vs. 41.2, *p* = 0.254), Zarit score (mean = 34.2 vs. 46.3, *p* = 0.142), or duration of social withdrawal (31.6 vs. 39.2, *p* = 0.264). However, subjects with missing values were significantly older than the rest (mean age 45.7 vs. 32.9, *p* = 0.002).

## Discussion

To our knowledge, this is the first study to investigate family factors related to social withdrawal syndrome in a culture other than Japanese. We found that social withdrawal had a high frequency of family psychiatric history, dysfunctional family dynamics, and traumatic events in childhood (family maltreatment). All of these factors were closely interrelated.

### Family Psychiatric History

Our study shows that there is a high frequency of family psychiatric history associated with social withdrawal syndrome, as pointed out in our previous study (2). Compared to one of the few epidemiological reports of family psychiatric history (40), the rates of family psychiatric history in our population were much higher than those of the general population. Cases with a family psychiatric history experienced earlier onset of isolation. In turn, they present more unfavorable family data, such as higher frequency of childhood maltreatment history and family dystocia, suggestive of the existence of dysfunctional family dynamics that make them a high-risk group for these factors as a whole.

It is known that children of parents with severe mental illness are at risk for a variety of psychiatric disorders and a third of them may develop a serious mental illness in early adulthood (41, 42). The most frequent family psychiatric disorders are affective and anxiety, the majority occurring in mothers. These results coincide with the few previous studies on family psychopathology and social withdrawal syndrome (6), as well as with other studies on maternal anxiety disorders and children with anxious school-refusal (43) and anxiety disorders in childhood (44). This association has been related to parenting behavior specific to mothers with anxiety disorders, in which mothers tend to reinforce the anxiety of children and their coping strategies of avoidance, and would interfere with their exploration and social activity. It could also be related to some hereditary genetic factors, requiring more research on this phenomenon.

In relation to the psychopathology of the father, our data reflect a higher frequency of psychotic and substance abuse disorders. Parental drug use disorder has been reported as a risk factor for depression, anxiety, social isolation, behavioral problems, and lower academic achievement (45–48). However, to our knowledge, this is the only study that has described the relationship between the psychopathology of the father and the offspring social withdrawal, which is not found in previous studies of social withdrawal syndrome (6).

An impairment in parenting has been reported in subgroups of parents with psychosis and low educational attainment, unemployment, poverty, and social isolation (49); the greater the difficulties in parenting, the greater the severity of the symptoms (50). These reports, together with our findings, point out impairment in parenting as a mediator in the association between parental mental disorder and risk factor for the development of the social withdrawal syndrome. This fact highlights the importance of prevention and early detection programs in this risk group (51) and the need for interventions to optimize successful parenting outcomes in the risk population. In addition, access to services and support programs by fathers is significantly lower than mothers (7), highlighting the need to emphasize aid programs for them.

### Dysfunctional Family Dynamics

In relation to family functioning, 61.5% of cases present family dystocia, demonstrating its high frequency, and coinciding with previous studies in which family function and social isolation syndrome have been related (6, 8, 10, 11, 52). Family dynamics, such as lack of problem-solving skills, poor communication, lack of emotional exchange in the family, and the difficulty of family members in sympathizing with each other's negative feelings, are attributed to the development of social withdrawal (8, 11). It is known that parenting styles, such as authoritarian, controlling, rejecting, and overprotective attitude, influence the development and stabilization of social withdrawal syndrome (6, 12, 53). It has been described that children with an absent father figure, together with a very close maternal bond, could experience difficulty in becoming independent adults (4, 23). Parental ambivalent attachment has been linked with social withdrawal syndrome (10). In turn, in the case of Japanese culture, hikikomori has been related to *amae*. Although the concept of *amae* was originally considered to be uniquely Japanese, recent opinions suggest that it is actually more universal and exists in other cultures (54, 55). In addition, especially as observed in social withdrawal syndrome, the development of basic interpersonal skills during the early stages of life seems to be insufficient, which could lead to vulnerability to stress in school/work environments and the tendency to escape from social situations (4, 23).

In our study, in 36 cases (18%), neither the patient nor the family allowed the CRHT to perform an initial in-home evaluation of the case. This phenomenon has been described previously and understood as an inability of the family to accept help, due to shame in revealing the problem and fear of symptom exacerbation or violence (4).

In addition, once home treatment had been started by our team, families with familial dystocia collaborated less (33.9 vs. 80.6%) with the therapeutic team in the guidelines and indications. This shows the difficulty and high stress that parents experience, their difficulty to intervene, their tendency to minimize or deny the problem, chronifying the situation for years without asking for help. In fact, in most cases (86%) the subjects with social withdrawal coexist with the family, which usually detects the problem and ultimately requests help (73.7%). Therefore, psychoeducation and family support for parents could help to lower their psychological distress, minimize the family's anxiety, solve the problems in family relationships, and consequently make a positive impact on the withdrawn patient (4, 7, 8).

### Family Maltreatment History

It is known that child maltreatment affects development and is related to various mental disorders, such as schizophrenia (56), bipolar disorder (56), and depressive and anxiety disorders (57). In relation to social withdrawal syndrome, the role of child maltreatment has been described as a risk factor for its development (6, 12, 19). In our study, we observed that 20.7% of cases have a history of maltreatment in childhood by the family, providing more evidence for the relationship of childhood abuse with social withdrawal syndrome, and confirming its role as a risk factor for development of social withdrawal. Cases with family abuse history present dysfunctional family dynamics more frequently, engage in less family collaboration with treatment, and are more likely to come from single-parent families. All of these factors contribute to an unfavorable familial environment, and probably increase the difficulty in detection, treatment, and clinical improvement of the syndrome. The data reinforce the need for effective programs and policies that reduce the occurrence of family abuse and facilitate early detection and treatment.

### Single-Parent Family

Almost 40% of cases live in a single-parent family, out of which 94% live with the mother. These data coincide with previous studies (5, 9) in which the predominance of the mother is suggested as a figure of coexistence and care of subjects with social isolation. Cases with single-parent families are younger and have less time for the evolution of withdrawal, maybe because the family previously sought help. These cases have less family psychiatric history, but more often show a history of childhood maltreatment and dysfunctional family dynamics, both factors that may be related to the fact that, at the time of consultation, the family has already fragmented and the mother has been the child's primary caretaker. Furthermore, single-parent cases have a higher frequency of hospital admissions after in-home treatment and higher Zarit scales scores, suggesting the difficulty in managing the social withdrawal of family at home.

## Strengths and Limitations

This study has several limitations and strengths. The main strength is that it is the first study in a culture other than Japanese, focusing on the family features related to social withdrawal syndrome, and providing more evidence on this phenomenon. One of the study's main limitations is not having used any objective measure by means of evaluation scales on variables, such as internet addiction, family dysfunctional dynamics, or the degree of family collaboration. In addition, the personal history, such as family abuse history, is provided by the subject or family member, without objective corroboration of the facts. Moreover, no information was collected on the time point and length of parental divorce, so it has not been possible to analyze whether it occurred before or during the period of isolation. The results of the multivariate regression model should be considered with caution as important correlation between the independent variables is known.

## Conclusions

Our study shows that negative family circumstances frequently accompany social withdrawal syndrome, and that most of them are related to isolation severity. These familial determinants of social isolation severity mainly co-occur rather than appear separately. Thus, community programs targeting social isolation should be designed to detect and evaluate each of these factors. Interventions targeting these familial determinants should be more cost-effective when offering a global family intervention rather than separate interventions for each factor, as more than one factor may co-occur, and mutual reinforcement may exist between some of them.

## Data Availability Statement

The datasets generated for this study are available on request to the corresponding author.

## Ethics Statement

This study was carried out in accordance with the recommendations of the Parc de Salut Mar Barcelona Clinical Research Ethical Committee. Anonymized data were extracted from the routine clinical follow-up performed in the Crisis Resolution Home Treatment, and no additional complementary examinations have been used or any intervention or treatment has been tested for this study. The main principles outlined in the Declaration of Helsinki were followed and the final protocol was approved by the Parc de Salut Mar Barcelona Clinical Research Ethical Committee.

## Author Contributions

ÁM-A and DB were involved in the initial conception, design of the study and development of the study protocol. ÁM-A, DC, MB, and AG were involved in the organization of logistics, recruitment of patients, and data collection and management. ÁM-A and DB were involved in the analysis and interpretation of data and wrote the manuscript. LM-L, VP, AB, and AT provided critical revision of the manuscript.

### Conflict of Interest

The authors declare that the research was conducted in the absence of any commercial or financial relationships that could be construed as a potential conflict of interest.
